# Functional Connectome Analyses Reveal the Human Olfactory Network Organization

**DOI:** 10.1523/ENEURO.0551-19.2020

**Published:** 2020-08-06

**Authors:** T. Campbell Arnold, Yuqi You, Mingzhou Ding, Xi-Nian Zuo, Ivan de Araujo, Wen Li

**Affiliations:** 1 Department of Psychology, Florida State University, Tallahassee, FL, 32306; 2 Department of Bioengineering, University of Pennsylvania, Philadelphia, PA, 19104; 3 J Crayton Pruitt Family Department of Biomedical Engineering, University of Florida, Gainesville, FL, 32611; 4 Developmental Population Neuroscience Research Center, State Key Laboratory of Cognitive Neuroscience and Learning & IDG/McGovern Institute for Brain Research, Beijing Normal University, Beijing, China, 100875; 5 Department of Psychiatry, Yale University School of Medicine, The John B. Pierce Laboratory, New Haven, CT, 06519; 6 Department of Neuroscience, Icahn School of Medicine at Mount Sinai, New York, NY, 10029

**Keywords:** functional neuroanatomy, functional segregation, functional specialization, graph theory, network, olfaction

## Abstract

The olfactory system is uniquely heterogeneous, performing multifaceted functions (beyond basic sensory processing) across diverse, widely distributed neural substrates. While knowledge of human olfaction continues to grow, it remains unclear how the olfactory network is organized to serve this unique set of functions. Leveraging a large and high-quality resting-state functional magnetic resonance imaging (rs-fMRI) dataset of nearly 900 participants from the Human Connectome Project (HCP), we identified a human olfactory network encompassing cortical and subcortical regions across the temporal and frontal lobes. Highlighting its reliability and generalizability, the connectivity matrix of this olfactory network mapped closely onto that extracted from an independent rs-fMRI dataset. Graph theoretical analysis further explicated the organizational principles of the network. The olfactory network exhibits a modular composition of three (i.e., the sensory, limbic, and frontal) subnetworks and demonstrates strong small-world properties, high in both global integration and local segregation (i.e., circuit specialization). This network organization thus ensures the segregation of local circuits, which are nonetheless integrated via connecting hubs [i.e., amygdala (AMY) and anterior insula (INSa)], thereby enabling the specialized, yet integrative, functions of olfaction. In particular, the degree of local segregation positively predicted olfactory discrimination performance in the independent sample, which we infer as a functional advantage of the network organization. In sum, an olfactory functional network has been identified through the large HCP dataset, affording a representative template of the human olfactory functional neuroanatomy. Importantly, the topological analysis of the olfactory network provides network-level insights into the remarkable functional specialization and spatial segregation of the olfactory system.

## Abstract (precis)

The olfactory system is uniquely heterogeneous, performing multifaceted functions (beyond basic sensory processing) across diverse, widely distributed neural substrates. While knowledge of human olfaction continues to grow, it remains unclear how the olfactory network is organized to serve this unique set of functions.

## Significance Statement

Olfaction is an intriguing multifunctional system, playing key roles in regulating emotions, autonomic tone, and feeding, beyond basic sensory perception. However, it is unclear how the neuroanatomy of olfaction is organized in humans to subserve these functions. Functional connectivity analysis of the Human Connectome Project (HCP) dataset combined with graph theoretical analysis revealed an optimized large-scale network consisting of three subnetworks, the sensory, limbic, and frontal subnetworks. Distributed across frontal and temporal lobes in well segregated fashion, these olfactory structures are also highly integrated, linked through hub nodes of the amygdala (AMY) and anterior insula (INSa). Our independent dataset replicated the HCP-derived olfactory network and, importantly, highlighted a direct association between the degree of network segregation and olfactory perception.

## Introduction

The olfactory system is uniquely heterogeneous, with functions that extend well beyond basic sensory processing to include domains of emotion, neuroendocrine, and homeostasis (Shipley and Ennis, 1996; Shepherd, 2004). Accordingly, the olfactory neuroanatomy involves widely distributed cortical and subcortical structures, exhibiting a high degree of functional specialization and spatial segregation (Kjelvik et al., 2012; Mainland et al., 2014; Kondoh et al., 2016; Zou et al., 2016). The human olfactory system comprises a set of primary (receiving direct bulbar input) and secondary olfactory regions in the temporal and frontal lobes (Carmichael et al., 1994; Gottfried and Zald, 2005; Zelano and Sobel, 2005; Seubert et al., 2013). Complex, large-scale networks integrated across distributed structures have been increasingly recognized as the fundamental organizational architectures and operational units of the brain (Varela et al., 2001; Fox and Raichle, 2007; Yuste, 2015). Here, we sought a network-level understanding of the human olfactory system, how are the olfactory regions organized to support diverse, yet highly integrated, functions?

Resting-state functional magnetic resonance imaging (rs-fMRI) in humans has revealed robust interregional coupling of spontaneous fMRI signal fluctuations underlying intrinsic functional connections (Biswal et al., 1995). This research has identified stable large-scale resting-state networks (RSNs), including networks of the physical (visual, auditory, and somatosensory) senses, but the olfactory network remains elusive. The largely subcortical composition of the olfactory system, with many loci at the air-tissue interface, has presented a serious challenge to olfactory network identification, especially for unguided, whole-brain rs-fMRI connectivity analysis. However, important insights into the olfactory network have been gained by targeting olfactory regions of interest (ROIs; Plailly et al., 2008; Royet et al., 2011; Krusemark and Li, 2012; Meunier et al., 2014; Kollndorfer et al., 2015; Novak et al., 2015; Sunwoo et al., 2015; Karunanayaka et al., 2017; Milardi et al., 2017; Ripp et al., 2018; Cecchetto et al., 2019; Zhou et al., 2019), especially in combination with network-science analysis (Royet et al., 2011; Meunier et al., 2014; Ripp et al., 2018; Zhou et al., 2019). The olfactory ROIs are fairly reliably identified, but inconsistencies in network composition and connections also abound in this literature (Fjaeldstad et al., 2017; Cecchetto et al., 2019).

Disparities in tasks employed in previous studies present a major source of inconsistency by engaging different regions and pathways. In comparison, rs-fMRI connectivity analysis is fairly immune to such confounds and has indeed revealed more reliable and robust connections than task-positive analyses (Sporns et al., 2000; Braun et al., 2012; Cao et al., 2014; Zuo et al., 2019). Another major source of inconsistency concerns the idiosyncratic nature of human olfactory perception and neuroanatomy (Richardson and Zucco, 1989; Krusemark and Li, 2012). That is, to produce a reliable and representative depiction of the system, sufficiently large samples are required to overcome individual variability, but the extant studies have been of modest sample sizes. To address these issues, we leveraged an extraordinary rs-fMRI dataset from the Human Connectome Project (HCP), consisting of nearly 900 individuals from diverse ethnicity/race (Van Essen et al., 2013), and combined ROI-based and whole-brain RS connectivity analysis to delineate the human olfactory network. Furthermore, to demonstrate the generalizability and reproducibility of this delineation, we repeated the analysis in an independent sample from our laboratory.

After defining the olfactory network composition, we attained insights into the functional organization of the olfactory network using meso-scale network analysis (Fortunato, 2010; Meunier et al., 2010; Karrer and Newman, 2011). Graph theory analysis represents a chief model for meso-scale network architecture (Bullmore and Sporns, 2009; Power et al., 2011) and was applied to explicate the organizational principles of the olfactory network. Importantly, using an olfactory discrimination task in the independent sample, we examined whether the level of network optimization would correlate with olfactory perception. In sum, we took three evolving steps here: (1) constructing an olfactory network based on functional connectivity strength; (2) defining the organization of the network and its functionality based on graph-theoretical analysis; and (3) linking individual levels of network optimization (i.e., small-world-ness) to olfactory performance by correlating graph metrics and discrimination accuracy.

## Materials and Methods

### Main study (the HCP dataset)

#### Participants

Participants for the main study were obtained from the open access HCP S900 data release (Van Essen et al., 2013). The full S900 release contains fMRI scans for 897 individuals; our analysis included the 812 subjects, who had all four resting-state scans and a voxel selection masks serving to remove low signal-to-noise ratio (SNR) voxels (Glasser et al., 2013). To ensure adequate and comparable fMRI signal strengths across the ROIs (many of which are located in areas that are highly susceptible to signal dropout and artifact), we further excluded participants who had (1) <50 voxels in any ROI (*n *=* *2); (2) a majority (>60%) of voxels in an anatomic ROI missing from the functional scans (*n *=* *57); or (3) a SNR of any ROI that was 3 standard deviations (SD) below the sample mean (*n *=* *60). The olfactory tubercle, a very small structure in humans, was exempted from this exclusion. Based on these criteria, 84 participants were excluded, resulting in a final sample of 728 participants (405 females; age: 28.8 ± 3.7 years).

#### Image acquisition and preprocessing

Resting-state scans were collected on a 3T Siemens Skyra MRI scanner and 32-channel head coil using a gradient-echo echoplanar imaging (EPI) sequence. The imaging parameters were TR/TE = 720/33 ms; flip angle = 52°; field of view = 208 × 180 mm; matrix size = 104 × 90; slice thickness = 2.0 mm, 72 slices, 2.0-mm isotropic voxels; multiband factor = 8. Two runs (one right-left and one left-right phase coding) were collected on each of two consecutive days. Each run contained 1200 volumes. Note, due to high signal dropout in APC and orbitofrontal cortex (OFC) regions in the left-right phase-encoding runs, only the right-left phase-encoding runs were included for these regions. The large number of scans for the right-left encoding runs (*n *=* *2400) would nonetheless ensure sufficient data for connectivity analysis concerning these regions. A T1-weighted structural image was collected on day one (0.7-mm isotropic voxels).

All images were preprocessed according to the HCP minimal preprocessing pipeline including artifact removal, motion correction, fieldmap correction, high-pass filtering, and normalization to the Montreal Neurologic Institute (MNI) template. Full details on the HCP data set and the preprocessing pipeline can be found in the S900 release manual and in previously published overviews of the HCP procedures (Glasser et al., 2013; Smith et al., 2013). Further motion correction procedures in preparation for connectivity analysis are described below.

#### Brain parcellation

The brain was parcellated based on a version of the Automated Anatomical Labeling (AAL) atlas (Tzourio-Mazoyer et al., 2002) that has been further subdivided into 600 cortical regions of roughly similar size through iterative bisection of larger regions (Hermundstad et al., 2013). Additionally, 28 cerebellum parcels were added (Diedrichsen et al., 2009). Several key olfactory regions, which are subcortical and/or not well defined in the AAL atlas, were drawn on the group-average anatomic T1 (HCP S900 release) in MRIcro (Rorden and Brett, 2000) in reference to a human brain atlas (Mai et al., 2008). These regions include the anterior and posterior piriform cortex (APC and PPC), amygdala (AMY), anterior and posterior hippocampus (aHIP and pHIP), entorhinal cortex (ENT), olfactory tubercle (OTB), nucleus accumbens (NAcc), and hypothalamus (HYP). The olfactory OFC (Oolf) region was defined by a 12 × 12 × 10 mm^3^ box centered around the putative Oolf centroid [25, 35, −14] (Gottfried and Zald, 2005). The eight insula parcels in the 600 parcellation set were merged into four regions, posterior, dorsal, ventral, and anterior insula (INSp, INSd, INSv, and INSa, respectively), to coincide with the human insular functional anatomy as delineated in a neuroimaging meta-analysis (Christopher et al., 2014). Voxels included in both the original AAL parcels and the drawn/adjusted regions were assigned to the latter, generating a final brain parcellation of 627 regions. The atlas was generated using the SPM (http://www.fil.ion.ucl.ac.uk/spm/software/spm12/) software packages for MATLAB (MathWorks) and FreeSurfer (Fischl, 2012).

The mean size of the parcels (excluding large cerebellum parcels) was 266 ± 76 voxels. To address signal dropout and artifacts, we conducted the following voxel removal procedure: (1) a brain mask generated from each participant’s T1 was used to exclude voxels located outside the participant’s brain; and (2) voxels determined to have a high coefficient of variation (COV) were excluded from the parcel (cutoff: COV > 0.5 SDs above the mean within a 5-mm σ Gaussian neighborhood; Glasser et al., 2013).

#### ROIs

**Figure 1.**
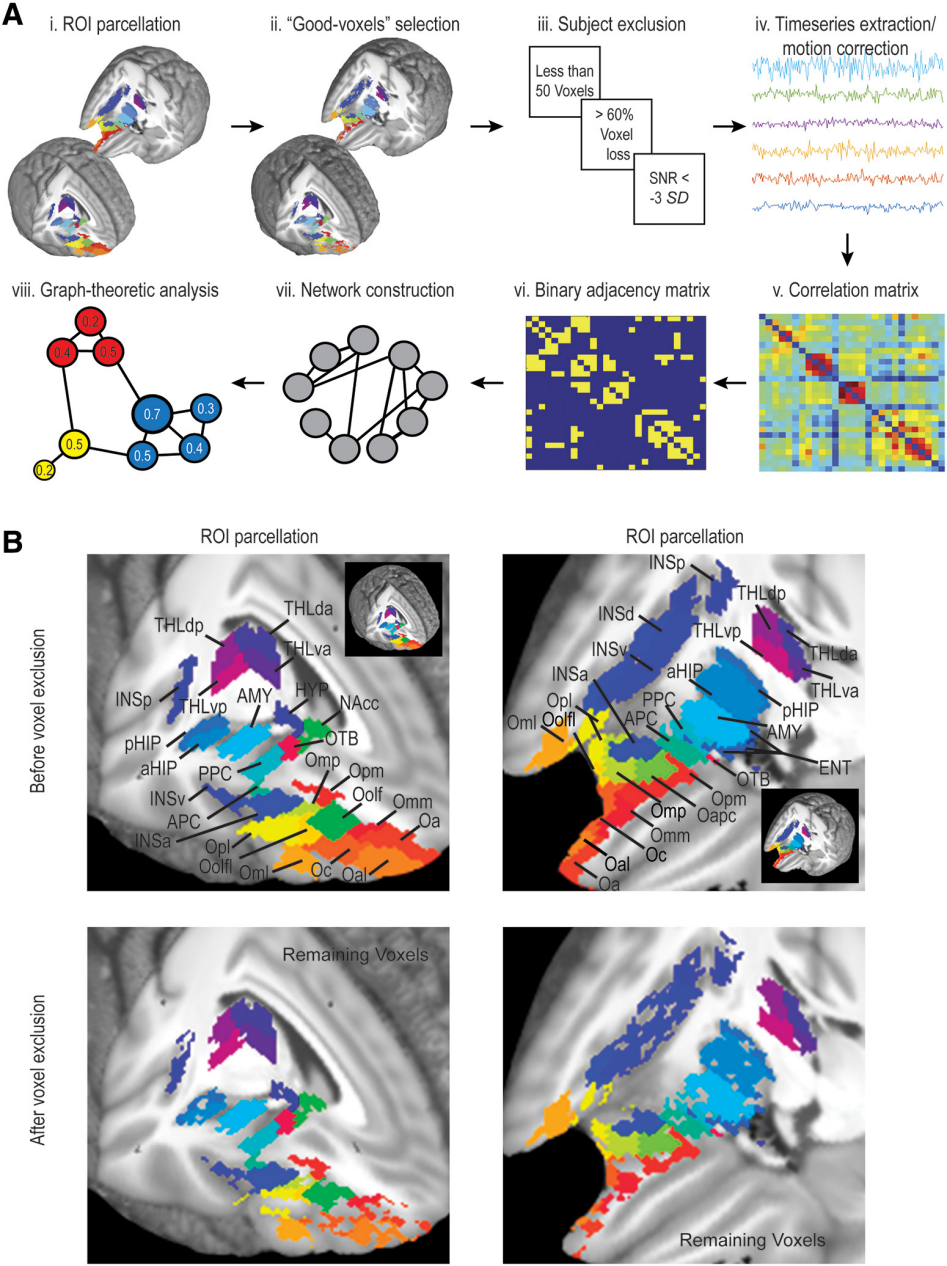
Procedures. ***A***, Schematic illustration of analysis pipeline. (i) A total of 28 ROIs were defined. (ii) An automated procedure based on COV removed voxels contaminated with artifacts from the ROIs. (iii) Participant exclusion based on three exclusion criteria. (iv) Timeseries data extraction from the ROIs. (v) A 28 × 28 correlation matrix compiled based on pair-wise correlations across the ROIs. (vi) A binary adjacency matrix constructed with suprathreshold and subthreshold connections. (vii) Suprathreshold connections chosen to form the olfactory network. (viii) Graph-theoretical analyses performed to characterize the organization of this network. ***B***, 3D display of ROIs before (top row) and after (bottom row) voxel removal. Insets illustrate the underlying ROIs in 3D whole-brain images with parts of dorsolateral frontal and temporal lobes removed. Omm, middle medial OFC; Opm, posterior medial OFC; Oc, central OFC; Oapc, anterior-APC OFC; Oml, medial lateral OFC; Oolfl = lateral olfactory OFC.

Twenty-eight regions were targeted as ROIs based on their possible roles in olfaction. (1) Key olfactory areas, five regions receiving direct olfactory bulbar input, including APC, PPC, AMY, ENT, and OTB (Carmichael et al., 1994; Haberly, 2001; Gottfried and Zald, 2005; Seubert et al., 2013), as well as the Oolf, a well-established region in the human olfactory neuroanatomy (Gottfried, 2010). The anterior olfactory nucleus was not included due to limited accessibility by fMRI. (2) Secondary, associative olfactory regions, additional regions in the OFC, insula (INSp, INSd, INSv, and INSa), thalamus [dorsal anterior thalamus (THLda), ventral anterior thalamus (THLva), dorsal posterior thalamus (THLdp), and ventral posterior thalamus (THLvp)], aHIP, pHIP, NAcc, and HYP (Carmichael et al., 1994; Haberly, 2001; Gottfried and Zald, 2005; Seubert et al., 2013). To have a comprehensive examination of the OFC participation in olfaction, we included the entire OFC (barring the most lateral parts), with a total of 11 parcels. The ventral medial prefrontal cortex was not considered here. Because of signal susceptibility at the tissue-air conjunction, we observed considerable signal dropout in the left hemisphere at the bottom of the frontal lobe and the fronto-temporal junction, affecting key ROIs (in the posterior OFC and APC). In light of predominantly ipsilateral olfactory pathways (Shipley and Ennis, 1996) and similar functional connectivity between the hemispheres (Zhou et al., 2019), we confined our olfactory network analysis to the right hemisphere where fMRI signals at the susceptible areas were well preserved. Table 1 shows centroid coordinates, sizes, and mean correlation coefficients (among the ROIs and among the whole brain) for all ROIs. Figure 1 illustrates the procedure (Fig. 1*A*) and anatomic locations of all ROIs before and after voxel removal Fig. 1*B*).

**Table 1:** ROI centroid coordinates, sizes, and mean (whole-brain) correlation coefficients

ROI	*x*	*y*	*z*	Voxels	*R*
Oolf	25	35	–14	180	0.25
APC	24	10	–20	143	0.22
PPC	24	4	–17	137	0.22
AMY	21	–5	–20	377	0.30
aHIP	26	–14	–21	312	0.28
pHIP	28	–28	–9	311	0.28
ENT	18	–4	–29	309	0.25
INSa	35	21	–6	449	0.30
INSd	39	8	6	421	0.27
INSv	43	2	–5	513	0.29
INSp	37	–15	12	368	0.29
HYP	5	–3	–12	107	0.22
THLda	11	–14	13	263	0.25
THLva	10	–13	2	276	0.27
THLdp	16	–24	11	238	0.24
THLvp	14	–25	2	280	0.27
OTB	14	4	–14	43	0.13
Omm	16	41	–21	204	0.24
Opm	17	25	–21	222	0.27
Oa	15	56	–17	250	0.23
Oc	28	43	–17	219	0.23
Oal	24	57	–14	252	0.20
Oml	42	38	–12	250	0.26
Oolfl	33	34	–15	183	0.24
Opl	39	26	–15	233	0.25
Omp	31	24	–21	160	0.22
Oapc	23	18	–23	134	0.22
NAcc	10	11	–8	116	0.22

#### Timeseries extraction and artifact removal

BOLD values for each scan were averaged across all voxels within an ROI for each resting-state run, resulting in four sets of 627 ROI timeseries each consisting of 1200 scans per participant. To further remove artifacts that could contribute to spurious resting-state (RS) activity variance (Fox et al., 2009; Power et al., 2012), several additional preprocessing steps were implemented. These steps included (1) mean centering and whitening of timeseries before concatenating runs of the same phase-encoding; (2) applying a temporal bandpass (0.01–0.08 Hz) filter (Biswal et al., 1995); (3) running a general linear model (GLM) to regress out head motion (Satterthwaite et al., 2013; Yan et al., 2013); the model contained 24 nuisance variables (Friston et al., 1996), including six head motion parameters from the current time point, the six parameters from the previous time point, and the squared values of the first twelve parameters; (4) and scrubbing of “spikes” containing significant motion based on framewise displacement index (FDi) defined as [FDi = ∣Δdix∣ + ∣Δdiy∣ + ∣Δdiz∣ + ∣Δαi∣ + ∣Δβi∣ + ∣Δγi∣]. Scans with FDi over 0.5 mm were classified as spikes in movement and removed (Power et al., 2012; Spielberg et al., 2015).

#### Network construction

Based on the extracted timeseries, Pearson’s correlation coefficients were calculated for each ROI pair and used to construct a 28 × 28 correlation matrix for each participant. These correlation coefficients were then Fisher Z-transformed, and for each pair, the coefficient was submitted to a *t*-test against its global baseline connectivity. This global baseline connectivity was defined for each pair as the average correlation coefficient (Fisher-transformed) of the two regions with all other (625) regions in the whole brain. A correlation in the top 5% of the 625 connections for each node was considered as significant (Watrous et al., 2013; Schedlbauer et al., 2014). A binary adjacency matrix, denoting suprathreshold and subthreshold connections, was thus constructed. These procedures are illustrated in Figure 1*Av*,*vi*.

For network construction, we first identified suprathreshold connections among the six key olfactory areas (APC, PPC, AMY, ENT, OTB, and Oolf). Secondary regions (i.e., regions receiving direct input from the primary regions) were defined as any of the remaining 22 ROIs that had at least one connection to one of the key olfactory regions and were thus admitted into the olfactory network.

#### Graph-theoretic analysis

To characterize the olfactory network, graph theory-based analysis was performed on the olfactory network constructed above using the brain connectivity toolbox (Rubinov and Sporns, 2010).

*Modularity*. Modularity maximization algorithms seek to find a division of nodes into subnetworks, or modules, which maximizes the number of intramodule connections while minimizing the number of intermodule connections (Newman, 2006). We performed modularity analysis using the Louvain algorithm (Blondel et al., 2008) as well as the Girvan–Newman algorithm (Girvan and Newman, 2002) with 10,000 iterations. Modularity index (*Q*) ranges [−1/2,1], with a cutoff score of 0.3 to indicate strong existence of subnetworks (Girvan and Newman, 2002). The reliability of modularity was further tested against randomly reassigned connections (*Q*_rand_) based on 10,000 permutations. This distribution was used to calculated the Z-scored modularity, with a score >3 indicating the existence of subnetworks (Fortunato, 2010; Kinnison et al., 2012). The network topology was then illustrated using the Gephi software with the Force Atlas and the expansion tool to increase spacing (Bastian et al., 2009; Zuo et al., 2012).

*Metrics of network functionality*. Several other graph theory metrics, concerning global and local qualities, were extracted to characterize the olfactory network. Small-world network organization is deemed to be highly efficient for spreading information and conserved across species (Rubinov and Sporns, 2010; Bota et al., 2015; Betzel and Bassett, 2018). We thus assessed small-world properties of the olfactory network by extracting two key markers, the global efficiency (*G*, indexing network integration and global communication; Latora and Marchiori, 2001) and clustering coefficient (*C*, indexing segregation or presence of local clusters; Watts and Strogatz, 1998). These indices were further contrasted with the average for 10,000 random reassignments of the edges in the network (*G*_rand_ and *C*_rand_). In a small-world network, global efficiency should be close to that of a random network characterized by a multitude of (random) connections whereas the clustering coefficient should be higher than that of a random network (as random connections are less likely to form clusters).

We also identified local regions key to the organization of the olfactory network. First, we assessed each region’s critical contribution to the network using targeted node deletion. We iteratively removed each of the regions (nodes) and examined the percentage reduction in global efficiency of the network (Bassett and Bullmore, 2006; van den Heuvel and Sporns, 2013). Second, we evaluated each region’s centrality (also known as “hubness”) in the network using three key metrics of centrality, node degree, betweenness centrality, and closeness centrality (Bota et al., 2015). These measures were further aggregated to generate an overall ranking score and a composite score by averaging across the z-scored values (Sporns et al., 2007). Node degree refers to the number of direct connections between a node and any other nodes in the network. Betweenness centrality represents the number of shortest paths that travel through a given node (Freeman, 1978), calculated as the fraction of all shortest paths in the network that contain a given node. Closeness centrality is a measure of the distance from a node to other nodes, calculated as the reciprocal of the average path length between a node and all other nodes in the network (Freeman, 1978). Lastly, we determined whether a hub was a connector or provincial hub using the participation coefficient (*P*), which indexes the diversity of module connections of a given node (Guimerà and Amaral, 2005).

#### Control analyses: (dis)connectivity of the olfactory network with the occipital visual cortex

To ascertain the validity and specificity of the olfactory network, we constructed a binary connectivity matrix between the olfactory network regions and occipital visual cortices. The occipital lobe was parcellated into a total of 28 parcels located in the calcarine (eight parcels), cuneus (four parcels), superior occipital gyrus (four parcels), middle occipital gyrus (eight parcels), and inferior occipital gyrus (four parcels) as defined in the 600 region parcellation of the AAL atlas (Tzourio-Mazoyer et al., 2002). These regions were parcellated into approximately equal sizes by the same method described above (Brain parcellation).

### Validation and extension study (the independent dataset)

As a validation of the olfactory network defined by the large HCP dataset, we applied this olfactory network to an independent rs-fMRI dataset collected in our lab. To further link the olfactory network functionality to olfactory performance, we administered an olfactory discrimination task immediately after the rs-fMRI scan and correlated participants’ global small-world metrics of their olfactory network with their task performance.

#### Participants

Thirty-three healthy participants took part in the study in exchange for course credit or monetary compensation. All participants were right-handed with normal olfaction, which was determined based on participants’ self-reported sense of smell and objective assessment (including odor intensity and pleasantness ratings) during a lab visit. Individuals showing aberrant olfactory performance or with nasal infections/allergies were excluded from participating in the study. Participants were also screened for any history of severe head injury, psychiatric or neurologic disorders or current use of psychotropic medication. All participants provided informed consent to participate in the study, which was approved by the University of Wisconsin-Madison Institutional Review Board. One participant was excluded due to metal artifact, leaving 32 participants (13 males; age 19.9 ± 2.0 years, range 18–30) in the final sample.

#### Odor discrimination task

Following a rs-fMRI scan (detailed below), we administered a 2-alternative-forced-choice (2AFC) odor discrimination task, where five binary odor mixtures with systematically varying proportions of acetophenone (“almond,” 5% l/l diluted in mineral oil) and eugenol (“clove,” 18% l/l) were presented. The five odor mixtures contained acetophenone/eugenol ratios of 80/20%, 60/40%, 50/50%, 40/60%, and 20/80%, respectively. Concentrations for the two odorants were determined through careful piloting to ensure equivalent perceived intensity. Before the test, participants were presented with acetophenone and eugenol in their original concentrations, which were labeled as Odor A and Odor B (the order was counterbalanced across participants). During the task, participants sniffed an odor mixture and indicated “Odor A” or “Odor B” using a button press. There were 15 trials for each mixture, randomly intermixed with a stimulus onset asynchrony (SOA) of 14.1 ms.

Odor mixtures were delivered at room temperature using a sixteen-channel computer-controlled olfactometer (airflow set at 1.5 l/min). When no odor was being presented, a control air flow was on at the same flow rate and temperature. This design permits rapid odor delivery in the absence of tactile, thermal, or auditory confounds (Lorig et al., 1999; Krusemark et al., 2013; Novak et al., 2015). Stimulus presentation and response recording were executed using Cogent software (Wellcome Department of Imaging Neuroscience, London, United Kingdom) as implemented in MATLAB.

#### rsMRI

*Image acquisition*. All participants underwent a 10 min rs-fMRI scan (with eyes open and fixated on the central crosshair) before the odor discrimination task. Gradient-echo T2-weighted echoplanar images (255 scans) were acquired with blood oxygen level-dependent (BOLD) contrast on a 3T GE MR750 MRI scanner, using an eight-channel head coil with sagittal acquisition. Imaging parameters were TR/TE: 2350/20 ms; flip angle: 60°, field of view 240 mm, slice thickness 2 mm, gap 1 mm; in-plane resolution/voxel size 1.72 × 1.72 mm; matrix size 128 × 128. A high-resolution (1 × 1 × 1 mm^3^) T1-weighted anatomic scan was also acquired. Lastly, a field map was acquired with a gradient echo sequence.

*Image analysis*. Imaging data (after removal of the first six dummy scans) were preprocessed in SPM12 (http://www.fil.ion.ucl.ac.uk/spm/software/spm12), including slice-time correction, spatial realignment, field-map correction, and normalization to MNI template (2 × 2 × 2 mm voxels) using diffeomorphic anatomical registration through exponentiated lie algebra (DARTEL; Ashburner, 2007). Based on the olfactory network identified through the HCP data, we focused on the 22 ROIs in the olfactory network, which were defined in the main study. We then applied the same artifact and voxel removal steps as described above (Timeseries extraction and artifact removal).

#### Correlation matrix and graph-theoretic metrics (weighted)

To examine individual-level graph metrics and associate them to individual differences in olfactory discrimination, we applied weighted graph theoretic analysis. Specifically, we calculated correlation coefficients for timeseries of any two regions, generating a correlation matrix (22 × 22) for each individual participant. We used the absolute value of correlations to reflect connectivity strength. The matrix was then multiplied with the binary matrix defined by the HCP dataset, constructing a sparse weighted matrix for each participant. The global graph metrics related to small-world-ness, the global efficiency and the clustering coefficient, were calculated based on this sparse weighted matrix (using absolute value weights).

#### Correlational analysis

Given the likely non-Gaussian distribution of correlation matrices, we computed the non-parametric Spearman correlation coefficient (ρ), and the statistical significance threshold was determined using null-model permutation tests (*n *=* *10,000; *p *<* *0.05 was set at 95th percentile). To assess reliability and generalizability of the HCP-based olfactory network, the group correlation matrix of the independent sample was correlated with the group correlation matrix of the HCP sample. We further examined the relationship between olfactory network organization and olfactory perceptual performance. To index basic olfactory discrimination, we applied signal detection theory analysis on the 2AFC performance and extracted *d*’ (Z_hit_ − Z_false alarm_) based on their responses to the dominantly acetophenone (80/20%) and dominantly eugenol (20/80%) mixtures. Each participant’s *d*’ score and their graph metrics (global efficiency and clustering coefficient) were then entered into Spearman correlational analysis.

#### Code accessibility

The code/software described in the paper is freely available online at https://github.com/LiLabFSU/olfactoryRSN. A description of each file is also available at https://github.com/LiLabFSU/olfactoryRSN. The code was run using a Linux subsystem on a Windows Server.

## Results

### The olfactory functional network

**Figure 2.**
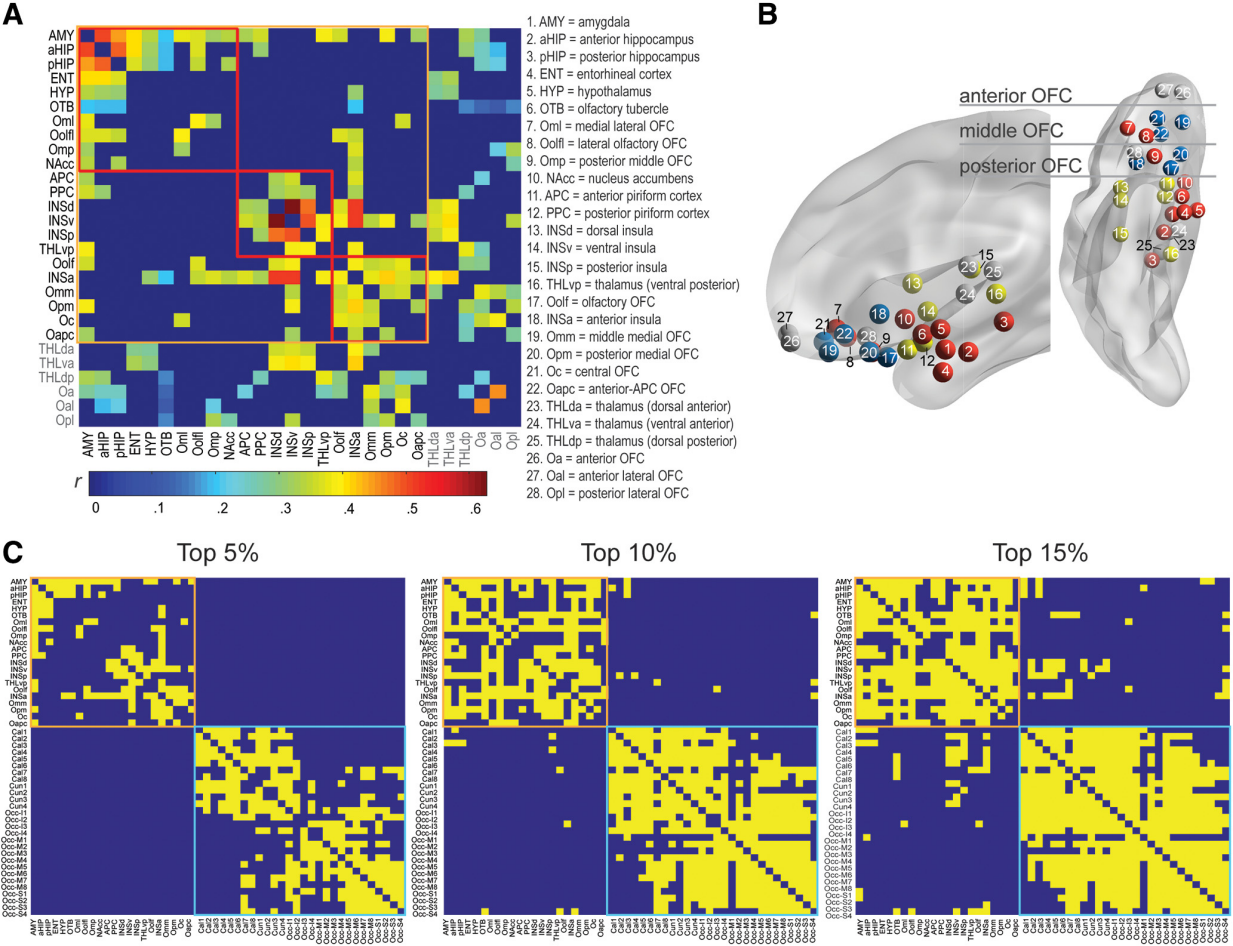
The olfactory network. ***A***, A weighted sparse 28 × 28 correlation matrix of group average Pearson’s *r*s for all suprathreshold pairs. ROIs included in the olfactory network are enclosed in the orange box, with the three identified modules (subnetworks) enclosed in the red boxes. The table lists the region names in correspondence to the ROI/node numbers. ***B***, A transparent brain model (in sagittal and axial views) with ROIs (nodes) for the three modules coded in three respective colors. Gray nodes are ROIs not accepted into the olfactory network. ***C***, A binary connectivity matrix reveals suprathreshold connections (shown in yellow) across the olfactory network nodes (22 parcels, enclosed in the orange box) and occipital visual cortical regions (28 parcels, enclosed in the cyan box) at three cutoff levels (top 5%, 10%, and 15%). The visual regions (Cal = calcarine gyrus; Cun = cuneus gyrus; Occ-I = occipital inferior gyrus; Occ-M = occipital middle gyrus; Occ-S = occipital superior gyrus) were strongly interconnected and relatively disconnected from the olfactory nodes.

According to the network construction procedure described above, we identified the olfactory network consisting of the six key olfactory ROIs and 16 additional parcels showing suprathreshold connectivity with one of the key regions (Fig. 2*A*,*B*). These 16 regions included the NAcc, HYP, both aHIP and pHIP, all four parcels of the insular cortex (INSa, INSp, INSv, and INSd), THLvp, and seven additional OFC parcels (located in the posterior and middle OFC). As illustrated in the weighted binary connectivity matrix (Fig. 2*A*), these 22 regions of the network were closely connected, constituting 28.6% (66/231) of all possible pair-wise connections. By contrast, exhibiting high modality specificity, this olfactory network showed no suprathreshold intermodality connections with occipital visual areas at 5% and only sparse ones at connection densities set at 10% and 15% (Fig. 2*C*).

### Network organization and characteristics

**Figure 3.**
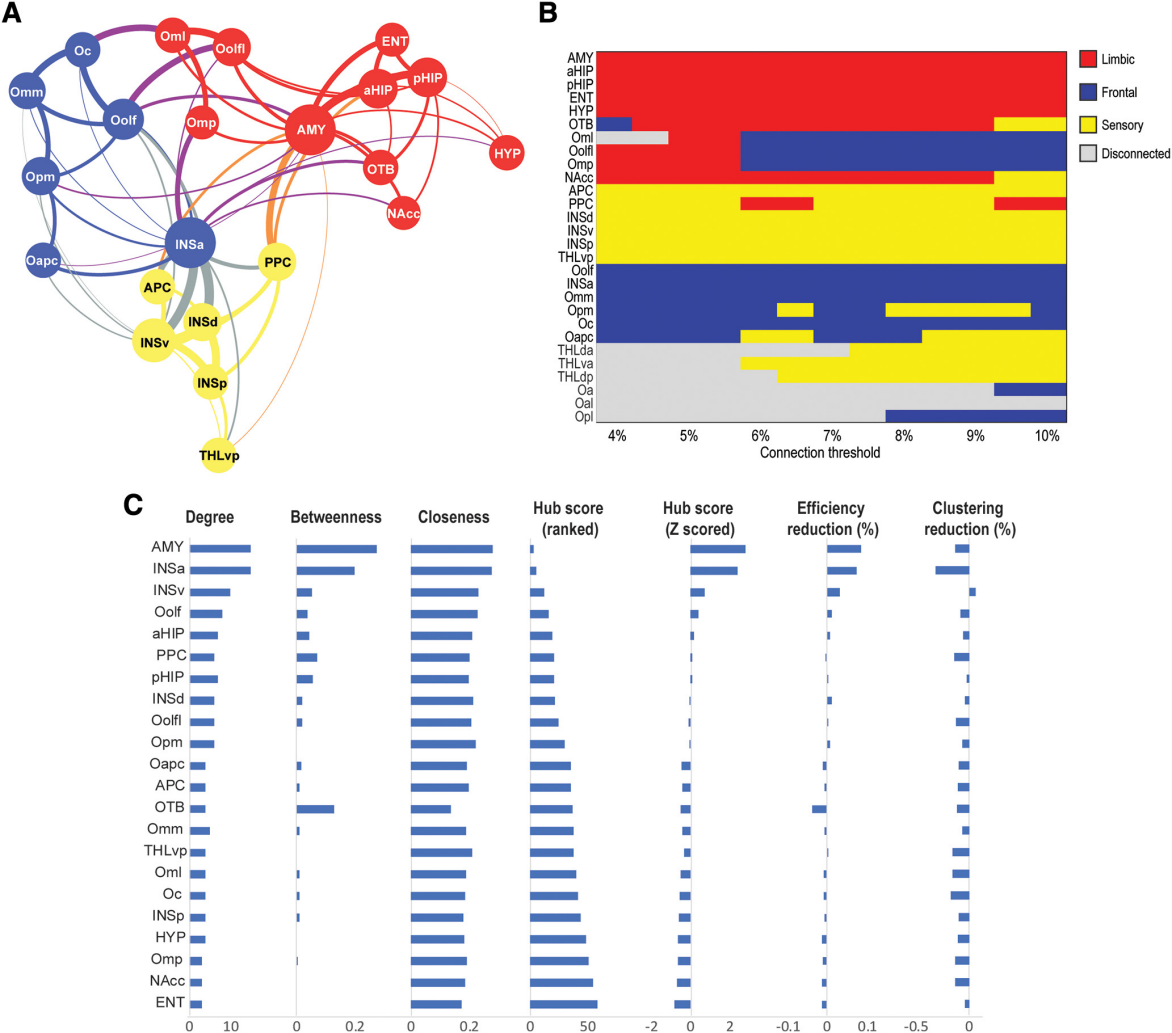
Local network metrics. ***A***, Topology of the olfactory network. The three modules/subnetworks are indicated by the three colors of the circles. Line thickness indicates connection strength (mean correlation coefficients), and node size reflects connection density (number of connections). ***B***, Modularity across a range of connection thresholds. Each row corresponds to one of the 28 ROIs, and columns indicate the connection threshold applied to the network while color indicates the module assignment. In general, nodes were consistently assigned to three modules identified as the limbic (red), sensory (yellow), and frontal (blue) subnetworks. At some connection thresholds, nodes were no longer connected to the network, which is indicated in gray. ***C***, Hubness of a node as reflected by composite hub ranking and composite hub Z-scores. The three centrality indices (node degree, betweenness, and closeness centrality) are also displayed. The AMY and INSa separated from the other nodes as major hubs of the network. Changes in global efficiency of the olfactory network following a node removal were small except for the AMY and INSa nodes, which resulted in 8.5% and 7.3% reductions in global efficiency, respectively.

Graph-theoretic modularity analysis revealed a strong modular organization of the olfactory network, with a reliable composition of three modules/subnetworks (*Q *= 0.30, *Z *=* *4.99, *p *<* *0.001; Fig. 3*A*). As illustrated in Figure 3*A*, the three modules/subnetworks could be characterized as (1) the “olfactory sensory subnetwork” consisting of APC, PPC, and three insular parcels (including the ventral, dorsal and posterior, but not the anterior, parcels), in addition to the ventral posterior thalamus on the periphery; (2) the “olfactory limbic/paralimbic subnetwork” consisting of the AMY, olfactory tubercle, and hippocampus at the center, in addition to the ENT, HYP, NAcc, and three OFC parcels on the periphery; and (3) the “olfactory frontal subnetwork” consisting of the INSa and Oolf at the center and four additional OFC parcels on the periphery. As indicated above, our conventional cutoff of 5% for significant connections showed a balance of sensitivity (i.e., a reasonable connectivity density of 28.6%) and specificity (no intermodality connections). To examine the stability of this network modularity, we then varied the threshold from 4% to 10% in 0.5% increments and observed a consistent three-module structure across these thresholds (Fig. 3*B*; Liang et al., 2016). We compared module partitions at each threshold to the partition at the 5% threshold and found a high degree of similarity (mean z-rand similarity = 0.82 ± 0.12; Traud et al., 2011).

These subnetworks were connected via multiple between-module connections. Based on a set of graph theoretic metrics of node centrality (i.e., degree, betweenness and closeness), the AMY and INSa stood out as two major hubs of the network (Fig. 3*B*), whose participant coefficient values (0.56 and 0.67, respectively) also indicated that they were connecting hubs between subnetworks (Fig. 3*A*). Graph-theoretic analysis further assessed small-world-ness of the olfactory network based on two defining features: global efficiency (*G*, indicating network integration and global communication) and clustering (*C*, indicating segregation of local clusters). Relative to random networks (based on 10,000 random permutations of the olfactory network connections) characterized by high efficiency but low clustering values, the olfactory network exhibited a high degree of small-world organization with high global efficiency (*G *=* *0.231, highly comparable to *G*_rand_ = 0.236) and high clustering (*C *=* *0.186 exceeding *C*_rand_ = 0.164). To exclude possible confounds in small-world analyses of the olfactory network (Bialonski et al., 2010; Papo et al., 2016), we further varied the connectivity threshold over the range of 4–10% and confirmed small-world-ness for the network at these thresholds (σ = 1.06 ± 0.05, σ > 1 indicates a small world network; Humphries and Gurney, 2008). We also note that our connectivity matrix was physiologically based (vs arbitrarily sampled over a grid system), and our network measures were normalized with degree preserving null models. Finally, we assayed the resilience of the olfactory network to local attacks using iterative node removal. The olfactory network sustained minimal impact by the removal of any one node (global efficiency change ranged −3.8% to 3.1%), with the exception of the hub regions (AMY and INSa) whose removal led to modest reductions (8.5% and 7.3%, respectively; Fig. 3*C*).

### Validating and linking network organization to olfactory perception in another sample

**Figure 4.**
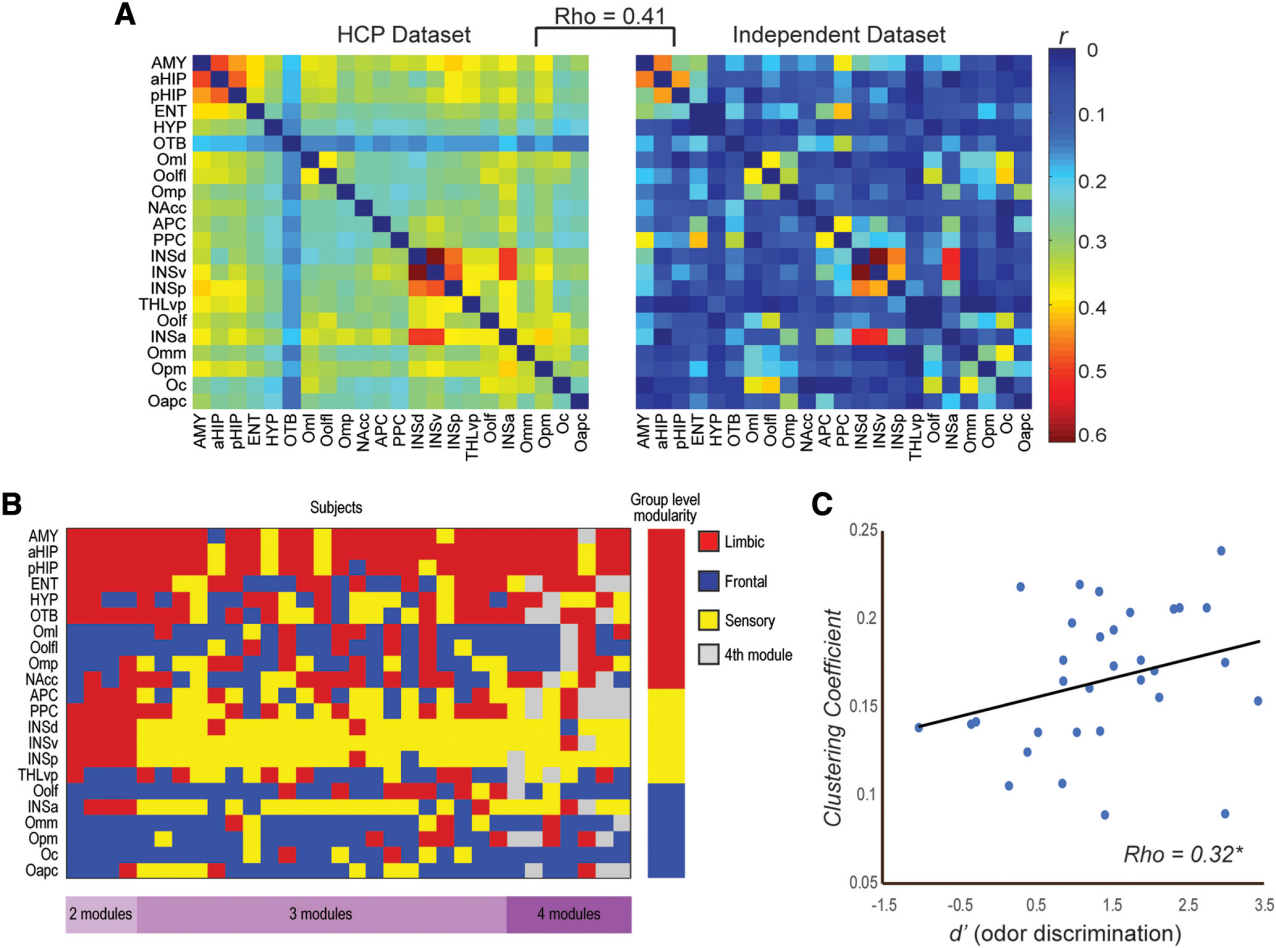
Validation and function of the olfactory network. ***A***, Weighted connectivity matrices of the olfactory network based on the HCP dataset and the independent dataset greatly overlapped; Spearman ρ = 0.41, *p *< 0.001. ***B***, Module assignments across weighted networks of individual subjects. Each row corresponds to one of the 28 ROIs, and columns indicate individual subjects while color indicates the module assignment. The group level module assignment is provided to the far right for reference. Subjects are ordered based on the number of modules detected (left, two module subjects, *n *=* *4; center, three module subjects, *n *=* *21; right, four module subjects, *n *=* *7) and beneath the module assignment matrix a key to module number is provided (purple). ***C***, The global network metric of clustering coefficient was positively correlated with olfactory discrimination performance (*d*’), ρ = 0.32, *p < 0.05.

To ascertain the functional relevance of the olfactory network organization, we then associated the olfactory network metrics with olfactory performance (i.e., odor discrimination) in an independent sample collected in the lab. First, we validated the olfactory network in this sample: there was strong concordance between the connectivity matrices derived from the HCP and independent samples (Spearman ρ* *=* *0.41, *p *<* *0.001; Fig. 4*A*), in support of the reliability and generalizability of the olfactory network. To demonstrate the reliability of this network structure, we then examined the concordance of each individual weighted network with the HCP-derived weighted network. We observed a high degree of concordance (Pearson’s *R *=* *0.42 ± 0.05). Figure 4*B* further illustrates a high degree of consistency in modular network formation across individual subjects, with the vast majority (*n *=* *21) exhibiting three-module networks comparable to the group-level network. Subject level modularity was significantly more similar to the group level partition than expected by random chance (mean z-rand similarity = 0.61, two-sampled *t* test, *p *<* *0.000001). A similarity analysis was also performed comparing all pairwise subject partitions to assess intersubject variability. We observed a high degree of agreement between subjects, which significantly exceeded chance values (mean z-rand similarity = 0.63, *p *<* *0.000001). Next, applying the olfactory network topology defined by the HCP dataset, we extracted each participant’s weighted small-world-ness metrics for the network, weighted global efficiency (*G*_w_) and clustering (*C*_w_). Spearman correlation analyses indicated that the odor discrimination performance, *d*’, correlated significantly with the clustering coefficient (ρ* = *0.32, *p *<* *0.05; Fig. 4*C*) but not global efficiency (ρ* = *0.13, *p* = 0.224).

We note that global metrics of certain networks are found to be associated with intelligence (Van Den Heuvel et al., 2009; Finn et al., 2015), which can modulate sensory processing (Melnick et al., 2013). However, the connectivity associated with intelligence is restricted to connectivity between frontal and parietal cortices, none of which were identified for the olfactory network, thereby unlikely to mediate the behavioral impact of the olfactory network. Compared with previous reports of strong associations between frontoparietal network efficiency and IQ (*r* ∼ 0.5), this impact of local segregation was of a medium strength. We suspect that noise in olfactory measurement (based on a single task) and fMRI susceptibility of olfactory regions could to some extent have weakened the observed association here.

## Discussion

Combining ROI and whole-brain analyses on the S900 HCP rs-fMRI dataset, we identified a human olfactory functional network of 22 interconnected parcels. Akin to the extraordinary size of the dataset that is conducive to high generalizability, this network demonstrated a strong concordance with the one extracted from our independent dataset. Graph theoretical analysis of the olfactory network further revealed an advantageous modular composition of three subnetworks, the sensory, limbic, and frontal subnetworks. The olfactory network also exhibited strong small-world properties, high in both global integration and local segregation. Importantly, the level of local segregation directly predicted odor discrimination performance in the independent sample. In sum, the current study provided a representative description of the human olfactory network and a template for the functional neuroanatomy of human olfaction. Furthermore, the network topology indicates an optimally organized architecture well suited for the diverse, specialized functions of olfaction.

This olfactory network comprised all ROIs (except for two anterior and one lateral OFC parcels and three thalamus parcels) implicated in rodent and non-human primate neuroanatomy, lending credence to the strong phylogenetic conservation of the olfactory system (Zelano and Sobel, 2005; Gottfried, 2010; Royet et al., 2011; Seubert et al., 2013). Connectivity density of the olfactory network was rather low (28.6%), which is consistent with other sensory networks (Young, 1993; Bassett and Bullmore, 2006) and accords with the evolutionary pressure to keep wiring to minimum to reduce communication cost (Cowey, 1979; Mitchison, 1991). Nonetheless, this connectivity density was in strong contrast with the highly sparse internetwork connectivity between the olfactory network and occipital visual cortices. That is, no internetwork connectivity survived the conventional statistical threshold (top 5%; Fig. 2*C*), confirming that the olfactory network exhibits strong modality specificity to maintain sensory fidelity.

The role of the thalamus in olfaction has been unclear in the literature. Here, the human olfactory network included only one parcel (in the ventral posterior portion) of the thalamus, which joined the network as a peripheral node. Furthermore, this parcel was connected only with the AMY and insula, known to receive strong thalamic projections largely conveying sensory input from other modalities (Augustine, 1996; Freese and Amaral, 2009; Gogolla, 2017). These results thus concur with the view of a minor contribution of the thalamus to the olfactory system (Smythies, 1997; Shepherd, 2005) and reinforce the notions of sparse thalamic connections with olfactory cortices and the lack of an obligatory thalamic relay (Price and Slotnick, 1983; Price, 1985). That said, while no corticothalamic connections reached the conventional threshold applied here (top 5%), they emerged at a lenient threshold (top 10%), including connections with the APC and multiple OFC parcels (Fig. 2*C*). Therefore, these findings permit the possibility that the weak corticothalamic pathways can be strengthened to become functionally relevant with certain task demands. For example, the olfactory-cortex-thalamus-OFC pathway was found to become significant during active attention, thereby engaging the OFC to subserve high-level olfactory processing (Plailly et al., 2008).

As the olfactory system supports not only olfactory sensory perception but also multiple non-sensory functions such as emotion and homeostasis (e.g., neuroendocrine regulation, reproductive response, feeding; Shipley, 1974), the composition of widely distributed cortical and subcortical structures in the olfactory network is consistent with the heterogeneous functions it serves. Accordingly, such diverse functions conducted across a widely distributed network would also demand a highly optimized network organization. Indeed, meso-scale graph theoretical analysis of the network topology confirmed the efficient organization of the olfactory network to suit its remarkable functionality.

First, we observed that the olfactory network had a strong degree of modularity, consisting of three modules with dense intramodule and loose intermodule connections. Modularity is a hallmark feature of optimized networks as formations of self-contained subdivisions effectively reduce overall network complexity and insulate local errors from global network functioning (Ash and Newth, 2007). Not only was the olfactory network compartmentalized into three subdivisions, but also the subdivisions were highly aligned with their diverse yet specialized functions. That is, a sensory subnetwork (comprising APC, PPC, and insula and thalamus parcels) would subserve basic olfactory sensory processing, a limbic subnetwork (comprising limbic ROIs and three lateral OFC parcels) would support emotion and homeostasis, and a frontal subnetwork (comprising OFC parcels and INSa) would underpin higher-level, integrative processes.

Second, the olfactory network had a “small-world” quality, a defining feature of a highly optimized network. Specifically, the olfactory network possessed a combination of high global efficiency and high local segregation. As such, the olfactory network assumed efficient communication across the subnetworks to allow for integrative processing while maintaining sufficient segregation to preserve specialized analysis. Evidently, this balance of global integration and subnetwork segregation is well suited for the olfactory system to sustain its diverse but specialized functions in a highly integrated manner.

Third, the olfactory subnetworks were integrated via two key hub structures, the AMY and INSa, akin to their strong connections with temporal and frontal structures (Freese and Amaral, 2009). In fact, given their connections with an extensive web of brain regions, the AMY and insula have been recognized as central hubs of large-scale neural systems (van den Heuvel and Sporns, 2013; Bickart et al., 2014; Gogolla, 2017). As such, with the intimate participation of the AMY and insula, the olfactory network is well positioned to summon a high level of global integration. Functionally, the AMY can relay emotional and homeostatic signals and the insula interoceptive signals to the sensory subnetwork to imbue olfactory perception with rich emotional and homeostatic information. Dovetailing with this organization, alliesthesia, a sensory experience that closely depends on the internal physiological milieu, prevails in olfaction (Cabanac, 1971, 1979; Krusemark et al., 2013). For instance, depending on the level of metabolic energy reserve (hungry or satiated), a food odor, while maintaining its odor identity (as processed in the sensory subnetwork), would take on distinct biological and emotional qualities (appetizing/pleasant or unappetizing/unpleasant) by integrating emotional and physiological signals relayed by the AMY and insula.

Lastly, the olfactory network would be resilient from local attacks. Complex networks are known to be tolerant to random errors such that a local malfunction would not cause global network dysfunctions (Callaway et al., 2000; Crucitti et al., 2004). Likewise, as we observed here, the removal of a region (other than the hubs) from the olfactory network resulted in minimal loss (<5%) in global efficiency. Remarkably, to the extent that specific hub failures often result in substantial global deficiency in a network (Callaway et al., 2000; Crucitti et al., 2004), the olfactory network sustained only modest loss (<10%) in global efficiency (van den Heuvel and Sporns, 2011) with the removal of the AMY or INSa. This level of resilience could be especially valuable for maintaining the overall integrity of the olfactory network as many of its structures, including the hubs (AMY and insula), are susceptible to pathologic invasions by disorders such as Alzheimer’s disease (Herzog and Kemper, 1980; Braak and Braak, 1991; Mesulam, 2015). This resilience can thus explain the fact that early-stage or preclinical patients maintain largely preserved global olfactory functions despite various (and often discrete) perceptual impairments (Doty et al., 1987; Royet et al., 2001; Djordjevic et al., 2008; Li et al., 2010; Wilson et al., 2010).

The functional relevance of the olfactory network organization was evinced by our independent dataset although we note as a limitation that the RS recordings were relatively brief (10 min). Specifically, we correlated small-world indices with olfactory discrimination performance and observed that local segregation, but not global efficiency, was critical for accurate olfactory discrimination. This impact of local segregation was of a medium strength, accounting for ∼10% of the total variance in olfactory discrimination. High segregation improves local efficiency and promotes specialized processing within local circuits (Rubinov and Sporns, 2010). Given its heterogeneous composition and the wealth of non-sensory input it receives, it stands to reason that the olfactory network needs to impose a certain level of functional insulation to its sensory subnetwork, thereby ensuring sensory fidelity in basic olfactory perception. That is, odor quality processing in the sensory subnetwork can be insulated against non-sensory influences from the other subnetworks such that olfactory perceptual validity is preserved. Alternatively, leakage from the limbic and frontal subnetworks, especially in individuals with low local segregation, would infuse an odor with hedonic hues or cognitive biases, which dominate and even alter olfactory perception. By this extension, variability in local segregation of the olfactory network could represent a viable network account for the idiosyncrasy of human olfactory experiences.

## Conclusion

The current study provides a representative and reliable depiction of the human olfactory network, which can be applied in future research as an anatomic template. The pattern of connections across an extended set of regions can guide investigation of olfactory circuits in normal and abnormal olfactory processing; and the submodules, composed of distributed regions, can form collective (vs discrete, individual) ROIs (e.g., the olfactory sensory ROI vs APC or insula) to represent unified working units in olfaction. Furthermore, our graph theoretical analysis confers network-level insights into human olfactory neuroanatomy, highlighting an evolutionarily conserved, topologically organized large-scale network. The compartmentalization of subnetworks allows for the multifaceted and yet specialized functions of olfaction, supporting sensation, emotion, neuroendocrine, and homeostasis. Critically, the strong global network integration nonetheless welds the subnetworks to subserve integrated processes. Arising from this highly optimized network, are the complex, varied, and almost infinite smells that define human olfaction (Yeshurun and Sobel, 2010; McGann, 2017).

## Synthesis

Reviewing Editor: Cheryl Sisk, Michigan State University Decisions are customarily a result of the Reviewing Editor and the peer reviewers coming together and discussing their recommendations until a consensus is reached. When revisions are invited, a fact-based synthesis statement explaining their decision and outlining what is needed to prepare a revision will be listed below.

Reviewers agree that the study is well designed and carefully considered. Reviewers noted several strengths, including: (1) The study delineates a human olfactory network that incorporates an extended set of distributed areas. This network is poorly understood, and the present results shed light on the connectivity patterns of human olfaction. (2) The study involves a careful selection of regions, based on a wide variety of empirical findings. (3) The use of an independent sample to examine the generalizability and reproducibility of the approach, helps to delineate the olfactory network with greater reliability. (4) The rich dataset used imparts remarkable rigor to the study. The limitations noted by the reviewers were mostly conceptual, and several questions were raised about the analyses. Please address these comments in your revised manuscript.

Conceptual

1. The main hypothesis, “that the olfactory network is optimally organized to facilitate olfactory perception”, is problematic, as it is not clear what type of outcomes would support or refute the hypothesis. There is nothing wrong with exploratory or data-driven analyses and in the field of neuroimaging, much harm has in fact been done by creating the impression that results came out as exactly as hypothesized (and by reviewers and editors encouraging such practices), which is almost never the case. So, I would like to encourage the authors to state clearly the original analysis plan, whether it was hypothesis-driven or exploratory, and any modifications of this plan along the way.

2. My major issue is simply a conceptual one. That is: at the fundamental level, this paper does not clearly present something discretely novel. Yes, the olfactory system involves cortical and subcortical systems - this has been well established since the late 19th century. Yes, the olfactory system is a part of several subsystems (sensory, limbic, frontal) - we know that as well to some degree. Information is segregated yet connected via some regions (called hubs here) - this is known as well. I’d like the authors to expand upon the notion that this data set provides a representative template for human olfactory functional neuroanatomy. Also, please expand on precisely what “insights” in the network specialization and segregation this study provides. If not earth-shattering new, that is ok. More clearly outlining in straight-forward statements what is new would be very useful and, may even result in greater impact of this work since presently some statements of novelty and innovation are obscure.

3. Regarding use of an extensive set of regions, may those regions be involved in different types of functions, unrelated to olfactory perception/processing? How specific are these networks to olfaction? Please discuss how the issue of cortical non-specificity may introduce unwanted confounds, and how they can be resolved.

4. A less major issue is the inclusion of the data section on “neurodegenerative pathological invasion”. Iterative node removal approaches do not recapitulate disease conditions well since neuroD disorders aren‘t simply characterized by “local attacks”. Instead, the influence of neuroD disorders on physiology on olfactory regions is very much under assessed mechanistically. These should be accompanied with mechanistic investigations to support validity. I think for this reason, and the seemingly tangential aspect of this section in the results (it came out of nowhere), it is wise to omit this text from the paper.

Analyses

5. As the authors state, the olfactory system is highly susceptible to artifacts, and the authors use a 3-step method to minimize these problems. This method may be useful to other researchers who face the same issues. Has the method, or similar methods, been used before? If so, please cite these papers. Does it lead to a measurable improvement of data quality?

6. The authors report associations between odor discrimination performance and network clustering coefficient. Could the correlation be related to other confounds, rather than being associated with the intrinsic properties of the network in terms of olfactory processing? When data from multiple distributed areas are analyzed together, the analysis may capture other properties which are also highly dependent on network connectivity measures (e.g., clustering coefficient, global efficiency in the resting brain)? One example of such confound is intelligence aptitude, which is known to influence various aspects sensory processing (e.g., discrimination accuracy and speed; Melnick et al., Curr. Biol, 2013; van den Heuvel et al., J. Neurosc., 2009). Can the authors exclude such possible confounds?

7. Given the issue of possible confounders, can the authors comment on the low rho value (around 0.30) for the correlation analysis results (p. 18).

8. It seems the outcomes of network connectivity analyses all come down to whatever threshold an experimenter sets as the index for what is ‘connected’. It is apparent based upon the work by other groups that one can find 3 [or many more] (as herein), 2, or even just 1 ‘network’ depending upon how the experimenter modifies the algorithms. Could the authors more clearly define how that was selected and what reasoning that was herein? Is the connectivity of these 3 networks identified herein resilient to subtle adjustments in connectivity parameters the algorithm is seeking out?

9. One of the main points is the finding that the reconstructed network has small world properties. There has been a considerable discussion whether or not such findings are artifacts. Please refer to papers Bialonski, S., Horstmann, M. T., and Lehnertz, K. (2010). From brain to earth and climate systems: small-world interaction networks or not? Chaos 20:013134. doi: 10.1063/1.3360561, and an opinion piece Papo D, Zanin M, Martínez JH, Buldú JM. Beware of the Small-World Neuroscientist! Front Hum Neurosci. 2016 Mar 8;10:96. doi: 10.3389/fnhum.2016.00096. eCollection 2016. The authors should comment on why they don’t think their findings fell into the same pitfall.

10. I always worry about the stability of the graph reconstruction. Unless I missed something, the authors show only averaged network from 32 participants. How different were the graph reconstructions for individual subjects? Also, within one subject - if the time series were divided in two parts and the reconstruction performed separately on the two time series, how similar would be the graph reconstruction in terms of connectivity?

Minor

11. Sporns, Tononi & Edelman, 2000 reference in page 4 should be Sporns, Tononi and Edelman, 2000

12. 10 minutes for a resting state scan might be considered brief, and some labs use longer scans. This could be mentioned as a limitation in the discussion.

## References

[B1] Ash J, Newth D (2007) Optimizing complex networks for resilience against cascading failure. Phys A Stat Mech its Appl 380:673–683. 10.1016/j.physa.2006.12.058

[B2] Ashburner J (2007) A fast diffeomorphic image registration algorithm. Neuroimage 38:95–113. 10.1016/j.neuroimage.2007.07.007 17761438

[B3] Augustine JR (1996) Circuitry and functional aspects of the insular lobe in primates including humans. Brain Res Brain Res Rev 22:229–244. 10.1016/s0165-0173(96)00011-2 8957561

[B4] Bassett DS, Bullmore E (2006) Small-world brain networks. Neuroscientist 12:512–523. 10.1177/1073858406293182 17079517

[B5] Bastian M, Heymann S, Jacomy M (2009) Gephi: an open source software for exploring and manipulating networks. Third International AAAI Conference on Weblogs and Social Media, May 17–20, San Jose, California.

[B6] Betzel RF, Bassett DS (2018) Specificity and robustness of long-distance connections in weighted, interareal connectomes. Proc Natl Acad Sci USA 115:E4880–E4889. 10.1073/pnas.1720186115 29739890 PMC6003515

[B7] Bialonski S, Horstmann MT, Lehnertz K (2010) From brain to earth and climate systems: small-world interaction networks or not? Chaos 20:13134. 10.1063/1.3360561 20370289

[B8] Bickart KC, Dickerson BC, Barrett LF (2014) The amygdala as a hub in brain networks that support social life. Neuropsychologia 63:235–248. 10.1016/j.neuropsychologia.2014.08.013 25152530 PMC4981504

[B9] Biswal B, Zerrin Yetkin F, Haughton VM, Hyde JS (1995) Functional connectivity in the motor cortex of resting human brain using echo‐planar MRI. Magn Reson Med 34:537–541. 10.1002/mrm.1910340409 8524021

[B10] Blondel VD, Guillaume JL, Lambiotte R, Lefebvre E (2008) Fast unfolding of communities in large networks. J Stat Mech 2008:P10008 10.1088/1742-5468/2008/10/P10008

[B11] Bota M, Sporns O, Swanson LW (2015) Architecture of the cerebral cortical association connectome underlying cognition. Proc Natl Acad Sci USA 112:E2093–E2101. 10.1073/pnas.1504394112 25848037 PMC4413280

[B12] Braak H, Braak E (1991) Demonstration of amyloid deposits and neurofibrillary changes in whole brain sections. Brain Pathol 1:213–216. 10.1111/j.1750-3639.1991.tb00661.x 1669710

[B13] Braun U, Plichta MM, Esslinger C, Sauer C, Haddad L, Grimm O, Mier D, Mohnke S, Heinz A, Erk S, Walter H, Seiferth N, Kirsch P, Meyer-Lindenberg A (2012) Test-retest reliability of resting-state connectivity network characteristics using fMRI and graph theoretical measures. Neuroimage 59:1404–1412. 10.1016/j.neuroimage.2011.08.044 21888983

[B14] Bullmore E, Sporns O (2009) Complex brain networks: graph theoretical analysis of structural and functional systems. Nat Rev Neurosci 10:186–198. 10.1038/nrn2575 19190637

[B15] Cabanac M (1971) Physiological role of pleasure. Science 173:1103–1107. 10.1126/science.173.4002.1103 5098954

[B16] Cabanac M (1979) Sensory pleasure. Q Rev Biol 54:1–29. 10.1086/410981 379894

[B17] Callaway DS, Newman MEJ, Strogatz SH, Watts DJ (2000) Network robustness and fragility: percolation on random graphs. Phys Rev Lett 85:5468–5471. 10.1103/PhysRevLett.85.5468 11136023

[B18] Cao H, Plichta MM, Schäfer A, Haddad L, Grimm O, Schneider M, Esslinger C, Kirsch P, Meyer-Lindenberg A, Tost H (2014) Test-retest reliability of fMRI-based graph theoretical properties during working memory, emotion processing, and resting state. Neuroimage 84:888–900. 10.1016/j.neuroimage.2013.09.013 24055506

[B19] Carmichael ST, Clugnet M‐C, Price JL (1994) Central olfactory connections in the macaque monkey. J Comp Neurol 346:403–434. 10.1002/cne.903460306 7527806

[B20] Cecchetto C, Fischmeister FP, Reichert JL, Bagga D, Schöpf V (2019) When to collect resting-state data: the influence of odor on post-task resting-state connectivity. Neuroimage 191:361–366. 10.1016/j.neuroimage.2019.02.050 30818023

[B21] Christopher L, Koshimori Y, Lang AE, Criaud M, Strafella AP (2014) Uncovering the role of the insula in non-motor symptoms of Parkinson’s disease. Brain 137:2143–2154. 10.1093/brain/awu084 24736308 PMC4107733

[B22] Cowey A (1979) Cortical maps and visual perception: the Grindley memorial lecture. Q J Exp Psychol 31:1–17. 10.1080/14640747908400703 424501

[B23] Crucitti P, Latora V, Marchiori M, Rapisarda A (2004) Error and attack tolerance of complex networks. Phys A Stat Mech its Appl 340:388–394. 10.1016/j.physa.2004.04.031

[B24] Diedrichsen J, Balsters JH, Flavell J, Cussans E, Ramnani N (2009) A probabilistic MR atlas of the human cerebellum. Neuroimage 46:39–46. 10.1016/j.neuroimage.2009.01.045 19457380

[B25] Djordjevic J, Jones-Gotman M, De Sousa K, Chertkow H (2008) Olfaction in patients with mild cognitive impairment and Alzheimer’s disease. Neurobiol Aging 29:693–706. 10.1016/j.neurobiolaging.2006.11.014 17207898

[B26] Doty RL, Reyes PF, Gregor T (1987) Presence of both odor identification and detection deficits in Alzheimer’s disease. Brain Res Bull 18:597–600. 10.1016/0361-9230(87)90129-8 3607528

[B27] Finn ES, Shen X, Scheinost D, Rosenberg MD, Huang J, Chun MM, Papademetris X, Constable RT (2015) Functional connectome fingerprinting: identifying individuals using patterns of brain connectivity. Nat Neurosci 18:1664–1671. 10.1038/nn.4135 26457551 PMC5008686

[B28] Fischl B (2012) FreeSurfer. Neuroimage 62:774–781. 10.1016/j.neuroimage.2012.01.021 22248573 PMC3685476

[B29] Fjaeldstad A, Fernandes HM, Van Hartevelt TJ, Gleesborg C, Møller A, Ovesen T, Kringelbach ML (2017) Brain fingerprints of olfaction: a novel structural method for assessing olfactory cortical networks in health and disease. Sci Rep 7:42534. 10.1038/srep42534 28195241 PMC5307346

[B30] Fortunato S (2010) Community detection in graphs. Phys Rep 486:75–174. 10.1016/j.physrep.2009.11.002

[B31] Fox MD, Raichle ME (2007) Spontaneous fluctuations in brain activity observed with functional magnetic resonance imaging. Nat Rev Neurosci 8:700–711. 10.1038/nrn2201 17704812

[B32] Fox MD, Zhang D, Snyder AZ, Raichle ME (2009) The global signal and observed anticorrelated resting state brain networks. J Neurophysiol 101:3270–3283. 10.1152/jn.90777.2008 19339462 PMC2694109

[B33] Freeman LC (1978) Centrality in social networks conceptual clarification. Soc Netw 1:215–239. 10.1016/0378-8733(78)90021-7

[B34] Freese JL, Amaral DG (2009) Neuroanatomy of the primate amygdala In: The human amygdala, pp 3–42. New York, NY: Guilford Press.

[B35] Friston KJ, Williams S, Howard R, Frackowiak RSJ, Turner R (1996) Movement-related effects in fMRI time-series. Magn Reson Med 35:346–355. 10.1002/mrm.1910350312 8699946

[B36] Girvan M, Newman MEJ (2002) Community structure in social and biological networks. Proc Natl Acad Sci USA 99:7821–7826. 10.1073/pnas.122653799 12060727 PMC122977

[B37] Glasser MF, Sotiropoulos SN, Wilson JA, Coalson TS, Fischl B, Andersson JL, Xu J, Jbabdi S, Webster M, Polimeni JR, Van Essen DC, Jenkinson M, WU-Minn HCP Consortium (2013) The minimal preprocessing pipelines for the human connectome project. Neuroimage 80:105–124. 10.1016/j.neuroimage.2013.04.127 23668970 PMC3720813

[B38] Gogolla N (2017) The insular cortex. Curr Biol 27:R580–R586. 10.1016/j.cub.2017.05.010 28633023

[B39] Gottfried J (2010) Olfaction and its pleasures: human neuroimaging perspectives In: Pleasures of the brain, pp 125–145. New York, NY: Oxford University Press.

[B40] Gottfried JA, Zald DH (2005) On the scent of human olfactory orbitofrontal cortex: meta-analysis and comparison to non-human primates. Brain Res Brain Res Rev 50:287–304. 10.1016/j.brainresrev.2005.08.004 16213593

[B41] Guimerà R, Amaral LAN (2005) Cartography of complex networks: modules and universal roles. J Stat Mech 2005:P02001-1–P02001-13. 10.1088/1742-5468/2005/02/P02001 PMC2151742 18159217

[B42] Haberly LB (2001) Parallel-distributed processing in olfactory cortex: new insights from morphological and physiological analysis of neuronal circuitry. Chem Senses 26:551–576. 10.1093/chemse/26.5.551 11418502

[B43] Hermundstad AM, Bassett DS, Brown KS, Aminoff EM, Clewett D, Freeman S, Frithsen A, Johnson A, Tipper CM, Miller MB, Grafton ST, Carlson JM (2013) Structural foundations of resting-state and task-based functional connectivity in the human brain. Proc Natl Acad Sci USA 110:6169–6174. 10.1073/pnas.1219562110 23530246 PMC3625268

[B44] Herzog AG, Kemper TL (1980) Amygdaloid changes in aging and dementia. Arch Neurol 37:625–629. 10.1001/archneur.1980.00500590049006 7425886

[B45] Humphries MD, Gurney K (2008) Network “small-world-ness”: a quantitative method for determining canonical network equivalence. PLoS One 3:e0002051 10.1371/journal.pone.0002051 18446219 PMC2323569

[B46] Karrer B, Newman MEJ (2011) Stochastic blockmodels and community structure in networks. Phys Rev E Stat Nonlinear Soft Matter Phys 83:16107. 10.1103/PhysRevE.83.016107 21405744

[B47] Karunanayaka PR, Wilson DA, Tobia MJ, Martinez BE, Meadowcroft MD, Eslinger PJ, Yang QX (2017) Default mode network deactivation during odor–visual association. Hum Brain Mapp 38:1125–1139. 10.1002/hbm.23440 27785847 PMC5326664

[B48] Kinnison J, Padmala S, Choi JM, Pessoa L (2012) Network analysis reveals increased integration during emotional and motivational processing. J Neurosci 32:8631–8372. 10.1523/JNEUROSCI.0821-12.2012 PMC3400262 22699916

[B49] Kjelvik G, Evensmoen HR, Brezova V, Håberg AK (2012) The human brain representation of odor identification. J Neurophysiol 108:645–657. 22539820 10.1152/jn.01036.2010

[B50] Kollndorfer K, Fischmeister FPS, Kowalczyk K, Hoche E, Mueller CA, Trattnig S, Schöpf V (2015) Olfactory training induces changes in regional functional connectivity in patients with long-term smell loss. NeuroImage Clin 9:401–410. 10.1016/j.nicl.2015.09.004 26594622 PMC4590718

[B51] Kondoh K, Lu Z, Ye X, Olson DP, Lowell BB, Buck LB (2016) A specific area of olfactory cortex involved in stress hormone responses to predator odours. Nature 532:103–106. 10.1038/nature17156 27001694 PMC5094457

[B52] Krusemark EA, Li W (2012) Enhanced olfactory sensory perception of threat in anxiety: an event-related fMRI study. Chemosens Percept 5:37–45. 10.1007/s12078-011-9111-7 22866182 PMC3410736

[B53] Krusemark EA, Novak LR, Gitelman DR, Li W (2013) When the sense of smell meets emotion: anxiety-state-dependent olfactory processing and neural circuitry adaptation. J Neurosci 33:15324–15332. 10.1523/JNEUROSCI.1835-13.2013 24068799 PMC3782615

[B54] Latora V, Marchiori M (2001) Efficient behavior of small-world networks. Phys Rev Lett 87:198701. 11690461 10.1103/PhysRevLett.87.198701

[B55] Li W, Howard JD, Gottfried JA (2010) Disruption of odour quality coding in piriform cortex mediates olfactory deficits in Alzheimer’s disease. Brain 133:2714–2726. 10.1093/brain/awq209 20724290 PMC2948816

[B56] Liang X, Zou Q, He Y, Yang Y (2016) Topologically reorganized connectivity architecture of default-mode, executive-control, and salience networks across working memory task loads. Cereb Cortex 26:1501–1511. 10.1093/cercor/bhu316 25596593 PMC4785946

[B57] Lorig TS, Elmes DG, Zald DH, Pardo JV (1999) A computer-controlled olfactometer for fMRI and electrophysiological studies of olfaction. Behav Res Methods Instrum Comput 31:370–375. 10.3758/bf03207734 10495824

[B58] Mai JK, Paxinos G, Voss T (2008) Atlas of the human brain, Ed 3. Cambridge, MA: Academic Press.

[B59] Mainland JD, Lundström JN, Reisert J, Lowe G (2014) From molecule to mind: an integrative perspective on odor intensity. Trends Neurosci 37:443–454. 24950600 10.1016/j.tins.2014.05.005 PMC4119848

[B60] McGann JP (2017) Poor human olfaction is a 19th-century myth. Science 356:eaam7263 10.1126/science.aam7263 28495701 PMC5512720

[B61] Melnick MD, Harrison BR, Park S, Bennetto L, Tadin D (2013) A strong interactive link between sensory discriminations and intelligence. Curr Biol 23:1013–1017. 10.1016/j.cub.2013.04.053 23707433 PMC3702042

[B62] Mesulam M (2015) Principles of behavioral and cognitive neurology. New York: Oxford University Press.

[B63] Meunier D, Lambiotte R, Bullmore ET (2010) Modular and hierarchically modular organization of brain networks. Front Neurosci 4:200. 10.3389/fnins.2010.00200 21151783 PMC3000003

[B64] Meunier D, Fonlupt P, Saive AL, Plailly J, Ravel N, Royet JP (2014) Modular structure of functional networks in olfactory memory. Neuroimage 95:264–275. 10.1016/j.neuroimage.2014.03.041 24662576

[B65] Milardi D, Cacciola A, Calamuneri A, Ghilardi MF, Caminiti F, Cascio F, Andronaco V, Anastasi G, Mormina E, Arrigo A, Bruschetta D, Quartarone A (2017) The olfactory system revealed: non-invasive mapping by using constrained spherical deconvolution tractography in healthy humans. Front Neuroanat 11:32. 10.3389/fnana.2017.00032 28443000 PMC5385345

[B66] Mitchison G (1991) Neuronal branching patterns and the economy of cortical wiring. Proc Biol Sci 245:151–158. 10.1098/rspb.1991.0102 1682939

[B67] Newman MEJ (2006) Finding community structure in networks using the eigenvectors of matrices. Phys Rev E Stat Nonlinear Soft Matter Phys 74:36104. 10.1103/PhysRevE.74.036104 17025705

[B68] Novak LR, Gitelman DR, Schuyler B, Li W (2015) Olfactory-visual integration facilitates perception of subthreshold negative emotion. Neuropsychologia 77:288–297. 10.1016/j.neuropsychologia.2015.09.005 26359718 PMC4699288

[B69] Papo D, Zanin M, Martínez JH, Buldú JM (2016) Beware of the small-world neuroscientist Front Hum Neurosci 10:96. 10.3389/fnhum.2016.00096 27014027 PMC4781830

[B70] Plailly J, Howard JD, Gitelman DR, Gottfried JA (2008) Attention to odor modulates thalamocortical connectivity in the human brain. J Neurosci 28:5257–5267. 10.1523/JNEUROSCI.5607-07.2008 18480282 PMC2706104

[B71] Power JD, Cohen AL, Nelson SM, Wig GS, Barnes KA, Church JA, Vogel AC, Laumann TO, Miezin FM, Schlaggar BL, Petersen SE (2011) Functional network organization of the human brain. Neuron 72:665–678. 10.1016/j.neuron.2011.09.006 22099467 PMC3222858

[B72] Power JD, Barnes KA, Snyder AZ, Schlaggar BL, Petersen SE (2012) Spurious but systematic correlations in functional connectivity MRI networks arise from subject motion. Neuroimage 59:2142–2154. 10.1016/j.neuroimage.2011.10.018 22019881 PMC3254728

[B73] Price JL (1985) Beyond the primary olfactory cortex: olfactory-related areas in the neocortex, thalamus and hypothalamus. Chem Senses 10:239–258. 10.1093/chemse/10.2.239

[B74] Price JL, Slotnick BM (1983) Dual olfactory representation in the rat thalamus: an anatomical and electrophysiological study. J Comp Neurol 215:63–77. 10.1002/cne.902150106 6853766

[B75] Richardson JTE, Zucco GM (1989) Cognition and olfaction: a review. Psychol Bull 105:352–360. 10.1037/0033-2909.105.3.352 2660177

[B76] Ripp I, zur Nieden AN, Blankenagel S, Franzmeier N, Lundström JN, Freiherr J (2018) Multisensory integration processing during olfactory-visual stimulation—An fMRI graph theoretical network analysis. Hum Brain Mapp 39:3713–3727. 10.1002/hbm.24206 29736907 PMC6866557

[B77] Rorden C, Brett M (2000) Stereotaxic display of brain lesions. Behav Neurol 12:191–200. 10.1155/2000/421719 11568431

[B78] Royet JP, Croisile B, Williamson-Vasta R, Hibert O, Serclerat D, Guerin J (2001) Rating of different olfactory judgements in Alzheimer’s disease. Chem Senses 26:409–417. 10.1093/chemse/26.4.409 11369675

[B79] Royet JP, Morin-Audebrand L, Cerf-Ducastel B, Haase L, Issanchou S, Murphy C, Fonlupt P, Sulmont-Rossé C, Plailly J (2011) True and false recognition memories of odors induce distinct neural signatures. Front Hum Neurosci 5:65. 10.3389/fnhum.2011.00065 21811450 PMC3143719

[B80] Rubinov M, Sporns O (2010) Complex network measures of brain connectivity: uses and interpretations. Neuroimage 52:1059–1069. 19819337 10.1016/j.neuroimage.2009.10.003

[B81] Satterthwaite TD, Elliott MA, Gerraty RT, Ruparel K, Loughead J, Calkins ME, Eickhoff SB, Hakonarson H, Gur RC, Gur RE, Wolf DH (2013) An improved framework for confound regression and filtering for control of motion artifact in the preprocessing of resting-state functional connectivity data. Neuroimage 64:240–256. 10.1016/j.neuroimage.2012.08.052 22926292 PMC3811142

[B82] Schedlbauer AM, Copara MS, Watrous AJ, Ekstrom AD (2014) Multiple interacting brain areas underlie successful spatiotemporal memory retrieval in humans. Sci Rep 4:6431. 10.1038/srep06431 25234342 PMC4168271

[B83] Seubert J, Freiherr J, Djordjevic J, Lundström JN (2013) Statistical localization of human olfactory cortex. Neuroimage 66:333–342. 10.1016/j.neuroimage.2012.10.030 23103688

[B84] Shepherd GM (2004) The human sense of smell: are we better than we think? PLoS Biol 2:e146. 10.1371/journal.pbio.0020146 15138509 PMC406401

[B85] Shepherd GM (2005) Perception without a thalamus: how does olfaction do it? Neuron 46:166–168. 10.1016/j.neuron.2005.03.012 15848795

[B86] Shipley MT (1974) Presubiculum afferents to the entorhinal area and the Papez circuit. Brain Res 67:162–168. 10.1016/0006-8993(74)90308-4 4220028

[B87] Shipley MT, Ennis M (1996) Functional organization of olfactory system. J Neurobiol 30:123–176. 10.1002/(SICI)1097-4695(199605)30:1<123::AID-NEU11>3.0.CO;2-N 8727988

[B88] Smith SM, Beckmann CF, Andersson J, Auerbach EJ, Bijsterbosch J, Douaud G, Duff E, Feinberg DA, Griffanti L, Harms MP, Kelly M, Laumann T, Miller KL, Moeller S, Petersen S, Power J, Salimi-Khorshidi G, Snyder AZ, Vu AT, Woolrich MW, et al. (2013) Resting-state fMRI in the human connectome project. Neuroimage 80:144–168. 10.1016/j.neuroimage.2013.05.039 23702415 PMC3720828

[B89] Smythies J (1997) The functional neuroanatomy of awareness: with a focus on the role of various anatomical systems in the control of intermodal attention. Conscious Cogn 6:455–481. 10.1006/ccog.1997.0315 9479480

[B90] Spielberg JM, McGlinchey RE, Milberg WP, Salat DH (2015) Brain network disturbance related to posttraumatic stress and traumatic brain injury in veterans. Biol Psychiatry 78:210–216. 10.1016/j.biopsych.2015.02.013 25818631

[B91] Sporns O, Tononi G, Edelman GM (2000) Connectivity and complexity: the relationship between neuroanatomy and brain dynamics. Neural Netw 13:909–922. 10.1016/S0893-6080(00)00053-8 11156201

[B92] Sporns O, Honey CJ, Kötter R (2007) Identification and classification of hubs in brain networks. PLoS One 2:e1049. 10.1371/journal.pone.0001049 17940613 PMC2013941

[B93] Sunwoo MK, Cha J, Ham JH, Song SK, Hong JY, Lee JM, Sohn YH, Lee PH (2015) Olfactory performance and resting state functional connectivity in non-demented drug naïve patients with Parkinson’s disease. Hum Brain Mapp 36:1716–1727. 10.1002/hbm.22732 25640661 PMC6869102

[B94] Traud AL, Kelsic ED, Mucha PJ, Porter MA (2011) Comparing community structure to characteristics in online collegiate social networks. SIAM Rev 53:526–543. 10.1137/080734315

[B95] Tzourio-Mazoyer N, Landeau B, Papathanassiou D, Crivello F, Etard O, Delcroix N, Mazoyer B, Joliot M (2002) Automated anatomical labeling of activations in SPM using a macroscopic anatomical parcellation of the MNI MRI single-subject brain. Neuroimage 15:273–289. 10.1006/nimg.2001.0978 11771995

[B96] van den Heuvel MP, Sporns O (2011) Rich-club organization of the human connectome. J Neurosci 31:15775–15786. 10.1523/JNEUROSCI.3539-11.2011 22049421 PMC6623027

[B97] van den Heuvel MP, Sporns O (2013) Network hubs in the human brain. Trends Cogn Sci 17:683–696. 10.1016/j.tics.2013.09.012 24231140

[B98] van den Heuvel MP, Stam CJ, Kahn RS, Hulshoff Pol HE (2009) Efficiency of functional brain networks and intellectual performance. J Neurosci 29:7619–7624. 10.1523/JNEUROSCI.1443-09.2009 19515930 PMC6665421

[B99] Van Essen DC, Smith SM, Barch DM, Behrens TEJ, Yacoub E, Ugurbil K; WU-Minn HCP Consortium (2013) The WU-Minn human connectome project: an overview. Neuroimage 80:62–79. 10.1016/j.neuroimage.2013.05.041 23684880 PMC3724347

[B100] Varela F, Lachaux JP, Rodriguez E, Martinerie J (2001) The brainweb: phase synchronization and large-scale integration. Nat Rev Neurosci 2:229–239. 10.1038/35067550 11283746

[B101] Watrous AJ, Tandon N, Conner CR, Pieters T, Ekstrom AD (2013) Frequency-specific network connectivity increases underlie accurate spatiotemporal memory retrieval. Nat Neurosci 16:349–356. 10.1038/nn.3315 23354333 PMC3581758

[B102] Watts DJ, Strogatz SH (1998) Collective dynamics of ‘small-world’ networks. Nature 393:440–442. 10.1038/30918 9623998

[B103] Wilson RS, Leurgans SE, Boyle PA, Schneider JA, Bennett DA (2010) Neurodegenerative basis of age-related cognitive decline. Neurology 75:1070–1078. 10.1212/WNL.0b013e3181f39adc 20844243 PMC2942064

[B104] Yan CG, Cheung B, Kelly C, Colcombe S, Craddock RC, Di Martino A, Li Q, Zuo XN, Castellanos FX, Milham MP (2013) A comprehensive assessment of regional variation in the impact of head micromovements on functional connectomics. Neuroimage 76:183–201. 10.1016/j.neuroimage.2013.03.004 23499792 PMC3896129

[B105] Yeshurun Y, Sobel N (2010) An odor is not worth a thousand words: from multidimensional odors to unidimensional odor objects. Annu Rev Psychol 61:219–241. 10.1146/annurev.psych.60.110707.163639 19958179

[B106] Young MP (1993) The organization of neural systems in the primate cerebral cortex. Proc Biol Sci 252:13–18. 10.1098/rspb.1993.0040 8389046

[B107] Yuste R (2015) From the neuron doctrine to neural networks. Nat Rev Neurosci 16:487–497. 10.1038/nrn3962 26152865

[B108] Zelano C, Sobel N (2005) Humans as an animal model for systems-level organization of olfaction. Neuron 48:431–454. 10.1016/j.neuron.2005.10.009 16269361

[B109] Zhou G, Lane G, Cooper SL, Kahnt T, Zelano C (2019) Characterizing functional pathways of the human olfactory system. Elife 8:e47177. 10.7554/eLife.47177 PMC6656430 31339489

[B110] Zou LQ, van Hartevelt TJ, Kringelbach ML, Cheung EFC, Chan RCK (2016) The neural mechanism of hedonic processing and judgment of pleasant odors: an activation likelihood estimation meta-analysis. Neuropsychology 30:970–979. 10.1037/neu0000292 27195988

[B111] Zuo XN, Ehmke R, Mennes M, Imperati D, Castellanos FX, Sporns O, Milham MP (2012) Network centrality in the human functional connectome. Cereb Cortex 22:1862–1875. 10.1093/cercor/bhr269 21968567

[B112] Zuo XN, Xu T, Milham MP (2019) Harnessing reliability for neuroscience research. Nat Hum Behav 3:768–771. 10.1038/s41562-019-0655-x 31253883

